# Inflammatory Signatures in First-Episode Psychosis: Comparing Substance-Free First-Episode Psychosis and Methamphetamine-Induced Psychosis

**DOI:** 10.1192/j.eurpsy.2026.10636

**Published:** 2026-07-31

**Authors:** F. Kulacaoglu, I. Bostan Ozgunduz, Y. E. Yildirim, E. Uyar

**Affiliations:** Psychiatry, Bakirkoy Prof Dr Mazhar Osman Research and Training Hospital for Psychiatry, Neurology and Neurosurgery, Istanbul, Türkiye

## Abstract

**Introduction:**

First-episode psychosis (FEP) can occur in substance-free individuals or emerge in the context of methamphetamine use. Inflammation has been implicated in the pathophysiology of psychosis, but comparative data on systemic inflammatory markers between these two FEP populations remain limited.

**Objectives:**

We aimed to compare blood inflammatory and metabolic parameters between patients with substance-free FEP and patients with first-episode methamphetamine-induced psychosis.

**Methods:**

This cross-sectional study included 211 patients with substance-free FEP, 199 patients with methamphetamine-induced FEP, and 101 healthy controls. Laboratory measurements comprised white blood cell count (WBC), neutrophils, lymphocytes, monocytes, platelets, triglycerides, low-density lipoprotein (LDL), and high-density lipoprotein (HDL). In addition, several derived inflammatory ratios were calculated: neutrophil-to-lymphocyte ratio (NLR), platelet-to-lymphocyte ratio (PLR), monocyte-to-lymphocyte ratio (MLR), monocyte-to-HDL cholesterol ratio (MHR), systemic immune-inflammation index (SII), systemic inflammation response index (SIRI), and pan-immune-inflammation value (PIV). Group comparisons were performed using ANOVA and post-hoc analyses, with statistical significance set at p<0.05.

**Results:**

WBC and neutrophil counts were elevated in both substance-free and methamphetamine-induced first-episode psychosis groups compared to healthy controls (p<0.001). Lymphocyte counts were significantly higher in the methamphetamine-induced group than in the substance-free group (p=0.001).Triglyceride levels were increased and HDL levels were decreased in the methamphetamine-induced group relative to both substance-free psychosis patients and controls (p<0.001), whereas LDL levels were reduced in both psychosis groups compared to controls (p<0.001). Regarding inflammatory indices, substance-free FIP patients demonstrated higher NLR, MLR, SII, SIRI, and PIV values compared to methamphetamine-induced psychosis patients (all p<0.05). In contrast, MHR was significantly higher in the methamphetamine-induced psychosis group (p=0.032). In substance-free FEP group, the NLR was significantly higher in those with a disease duration of 1–5 years compared to first-year patients (p=0.020).

**Conclusions:**

Methamphetamine-induced FEP was associated with metabolic dysregulation (elevated triglycerides, low HDL, higher MHR), whereas substance-free FEP was characterized by a stronger systemic inflammatory response (elevated NLR, MLR, SII, SIRI, PIV). As a recognized marker of systemic inflammation, elevated NLR may indicate an increased inflammatory burden with longer illness duration These differential biomarker patterns suggest distinct underlying mechanisms and may aid in the clinical differentiation and prognostic evaluation of first-episode psychosis.

**Disclosure of Interest:**

None Declared

